# *GJA4*/Connexin 37 Mutations Correlate with Secondary Lymphedema Following Surgery in Breast Cancer Patients

**DOI:** 10.3390/biomedicines6010023

**Published:** 2018-02-22

**Authors:** Mahrooyeh Hadizadeh, Seiied Mojtaba Mohaddes Ardebili, Mansoor Salehi, Chris Young, Fariborz Mokarian, James McClellan, Qin Xu, Mohammad Kazemi, Elham Moazam, Behzad Mahaki, Maziar Ashrafian Bonab

**Affiliations:** 1 Department of Medical Genetics, Faculty of Medicine, Tabriz University of Medical Sciences, Tabriz 5166614766, Iran; mahrooyeh.hadizadeh@gmail.com (M.H.); Dr.mohades.lab@gmail.com (S.M.M.A.); 2 Department of Genetics and Molecular Biology, Isfahan University of Medical Sciences, Isfahan 81746753461, Iran; m_salehi@med.mui.ac.ir (M.S.); m_kazemi@med.mui.ac.ir (M.K.); 3 School of Allied Health Sciences, Faculty of Health and Life Sciences, De Montfort University, Leicester LE1 9BH, UK; chris.young@dmu.ac.uk; 4 Cancer Prevention Research Centre, Isfahan University of Medical Sciences, Isfahan 8184917911, Iran; mokarian@med.mui.ac.ir (F.M.); el_moazam@yahoo.com (E.M.); 5 School of Biological Sciences, University of Portsmouth, Portsmouth PO1 2DY, UK; chopsalotapepl@yahoo.co.uk; 6 School of Pharmacy, Faculty of Health and Life Sciences, De Montfort University, Leicester LE1 9BH, UK; qin.xu@dmu.ac.uk; 7 Department of Occupational Health Engineering, School of Health, Isfahan University of Medical Sciences, Isfahan 8174673461, Iran; behzad.mahaki@gmail.com; 8 Department of Biological Sciences, University of Chester, Chester CH1 4BJ, UK

**Keywords:** connexin 37, secondary lymphedema, breast cancer, *GJA4* gene, single nucleotide polymorphism (SNP)

## Abstract

Lymphedema is a condition resulting from mutations in various genes essential for lymphatic development and function, which leads to obstruction of the lymphatic system. Secondary lymphedema is a progressive and incurable condition, most often manifesting after surgery for breast cancer. Although its causation appears complex, various lines of evidence indicate that genetic predisposition may play a role. Previous studies show that mutations in connexin 47 are associated with secondary lymphedema. We have tested the hypothesis that connexin 37 gene mutations in humans are associated with secondary lymphedema following breast cancer surgery. A total of 2211 breast cancer patients were screened and tested for reference single nucleotide polymorphisms (SNPs) of the *GJA4* gene (gap junction protein alpha 4 gene). The results presented in this paper indicate that two SNPs in the 3’ UTR (the three prime untranslated region) of the *GJA4* gene are associated with an increased risk of secondary lymphedema in patients undergoing breast cancer treatment. Our results provide evidence of a novel genetic biomarker for assessing the predisposition to secondary lymphedema in human breast cancer patients. Testing for the condition-associated alleles described here could assist and inform treatment and post-operative care plans of breast cancer patients, with potentially positive outcomes for the management of disease progression.

## 1. Introduction

Connexins are a large family of six-subunit transmembrane hemi-channels. A total of 21 connexin genes have been described in humans, and 20 in mice [1,2]. Individual hemi-channels (connexons) as part of a gap junction channel allow for the diffusion of ions and small molecules between the extracellular space and the cytosol, and gap junction channels facilitate the diffusion of ions, metabolites, and signalling molecules between cells [3,4].

Lymphedema is an incurable condition resulting from obstruction of the lymphatic system, characterised by localised fluid retention, swelling, and susceptibility to infection. The condition is sub-classified into two varieties: the so-called primary lymphedema is inherited, resulting from mutations in various genes essential for lymphatic development and function, whilst secondary lymphedema is generally a post-operative complication of surgery, usually affecting women undergoing treatment for breast cancer [5,6,7]. The estimates of the proportion of patients affected range from 2–80%, no doubt partly reflecting differences in measurement and diagnostic criteria [2]. Several other medical factors, such as the stage of cancer at the time of diagnosis, the pathological involvement of lymph nodes, the number of dissected lymph nodes during breast cancer surgery, the type and extent of surgery, and also the extent and method of radio- and chemotherapy are considered important in the development of secondary lymphedema in breast cancer patients. Additionally, patient age, body mass index, and degree of physical activity have all been suggested to influence the risk of developing secondary lymphedema [7,8].

Intriguingly, there is also some evidence for genetic predisposition to secondary lymphedema [9,10]. For example, mutations in hepatocyte growth factor/high affinity hepatocyte growth factor receptor/mesenchymal-epithelial transition (*HGF*/*MET*) have been reported in both primary and secondary lymphedema [9]. This protein is expressed in lymphatic endothelial cells and has functions in cell growth, mobility, differentiation, and intercellular junctions [9]. Another set of mutations associated with secondary lymphedema affect the connexin Cx47 [8]. Similar mutations are also associated with Pelizaues–Merzbacher-like disease (PMLD) [11], spastic paraplegia [12], and primary lymphedema [11,12]. It has been shown that Cx43 is abundantly expressed in the ventricular myocardium and in cardiac neural crest cells and plays an important role in human congenital heart disease [13].

Connexins adopt complex tertiary structures achieved through the coordination of six subunits, representing a “connexon”, which is capable of generating a gap junction by docking to another connexon on an adjacent cell [14]. This suggests a general model in which a genetic predisposition to form inappropriate cellular junctions may explain the development of some secondary lymphedemas.

Here, we demonstrate that polymorphisms in another connexin, Cx37, are differentially distributed in patients with and without secondary lymphedema, following surgery for breast cancer. Cx37 is a good candidate marker because it is expressed in the lymphatic system and endothelial cells [15]. Furthermore, single nucleotide polymorphisms (SNPs) in *GJA4* (the gene that codes for Cx37) have previously been shown to be associated with myocardial infarction and atherosclerosis, suggesting (by analogy with the wide-ranging effects of mutations in *HGF*/*MET* and Cx47), that Cx37 could have a role in secondary lymphedema [16].

## 2. Experimental Section

### 2.1. Patients

From an initial screen of 2211 breast cancer patients (admitted to the Sayyed-Al-Shohada hospital in Isfahan, Iran, between 2009–2015) at least 6 months post chemotherapy, written consents were obtained and blood samples collected from 102 patients aged between 35 and 70. Patients were selected for this study if they had breast cancer “lower than stage IIIC” and “tumour size between 3 and 10 cm”. From the patients with the above characteristics, 51 patients with secondary lymphedema (case group) and 51 patients without secondary lymphedema (control group) were randomly selected and were further analysed. The staging system of the International Society of Lymphology (ISL) was used to characterize the severity of lymphedema, considering the “softness” or “firmness” of the limb, and all patients in the case group had moderate to severe lymphedema [17].

All 102 patients either had modified radical mastectomy (MRM) or breast conserving surgery (BCS). In the case group, an average of 4.7 and in control group an average of 2.2 lymph nodes were involved. Also, 69% of patients from the case group and 64% of patients from the control group had BCS, and 31% of patients from the case group and 46% of patients from the control group had MRM.

During the surgery, at least six axillary lymph nodes were removed, and the patients had chemotherapy and radiation therapy (supplementary material). The external beam radiation method was applied to all patients using a linear accelerator on an outpatient basis, 5 days a week, over 5 to 7 weeks, depending on each particular situation. The radiotherapy treatment included the breast and the regional axillary lymph nodes, and there was no clear correlation between the radiotherapy of regional lymph nodes and the occurrence of secondary lymphedema. DNA extraction from blood samples was performed using PrimePrep Genomic DNA isolation kit (*GeNet* Bio, Daejon, Korea) [18]. 

All subjects gave their informed consent for inclusion before they participated in the study. The study was conducted in accordance with the Declaration of Helsinki, and the protocol was approved by the Ethics Committee of the Tabriz University of Medical Sciences, Iran (IR.MUI.REC.1394.2.058 (1 August 2014)).

### 2.2. High-Resolution Melting Analysis

High-Resolution Melting (HRM) is an inexpensive, accurate, homogeneous, and post-PCR method, which enables researchers to analyse genetic variations such as SNPs, mutations, and methylations in PCR amplicons [19]. The primers for amplification of rs3543 and rs705193 were designed using Primer3web software (version 4.1.0, Howard Hughes Medical Institute, Ashburn, VA, USA; http://primer3.ut.ee/). The primer sequences and product sizes are shown in Table 1. PCR amplification and HRM analysis were performed in a reaction volume of 10 µL, with High-Resolution Master Mix (Solis BioDyne, Tartu, Estonia; https://www.sbd.ee/), 0.5 µL of each primer (10 pmol), and 30 ng DNA. HRM analysis was performed using a Corbett Rotor-Gene 6000 (Germantown, MD, USA).

The polymerase chain reaction (PCR) procedure started with a pre-incubation at 95 °C for 15 min, followed by 40 cycles of denaturation (95 °C for 15 s), annealing (60 °C for 20 s), and extension (72 °C for 20 s). The melting analysis of the amplicons was carried out from 75 °C to 95 °C at 0.2 °C/s. The samples with different melting profiles were selected for direct sequencing by an ABI 3130 sequencer (Applied Biosystems, Waltham, MA, USA; http://www.thermofisher.com).

### 2.3. Statistical Analysis

SPSS version 22 (IBM SPSS,) was used for all statistical analyses. Parametric analyses were conducted on continuous data with normal distribution, otherwise non-parametric analyses were applied. The significance level of *p* < 0.05 was used in each analysis.

## 3. Results

### 3.1. Physiological Parameters

Clinical records of patients’ age, height, weight (at the time of sampling and after surgery and radiotherapy), and body surface area were collected; the statistical analyses showed no significant differences between lymphedema case and control groups for these parameters (Table 2). The effects of the physiological parameters on the presence of secondary lymphedema were further evaluated using binary logistic regression (Table 3). The regression revealed no significant effects (Cox & Snell R^2^ = 0.040, Nagelkerke R^2^ = 0.053, *p* > 0.05) of these parameters on the presence of secondary lymphedema.

Age, height, and weight odds ratios were close to 1, indicating no effects (Table 2), while an increase in the body surface area did appear to correlate with an increased risk of secondary lymphedema, yet the effect was not statistically significant (*p* = 0.604).

### 3.2. Tumour Parameters

In the case group, 35 patients went through the MRM surgical procedure and 16 through the BCS procedure, while in the control group 28 patients underwent the MRM procedure and 23 the BCS procedure. There was no statistical significant difference between the two groups (Cramer’s V = 0.141, *p* = 0.154). The lymph nodes removed during surgery varied from patient to patient. The highest number of nodes removed in the control group was 20 and the lowest 6; 51% had 6–10 nodes removed. In comparison to the control group, the patients in the case group had a maximum of 28 lymph nodes removed (1 patient) and a minimum of 6; 66.7% had 6–10 nodes removed. However, the statistical analyses showed no significant differences in the number of lymph nodes removed between the two groups (Table 2).

**Figure 1:**
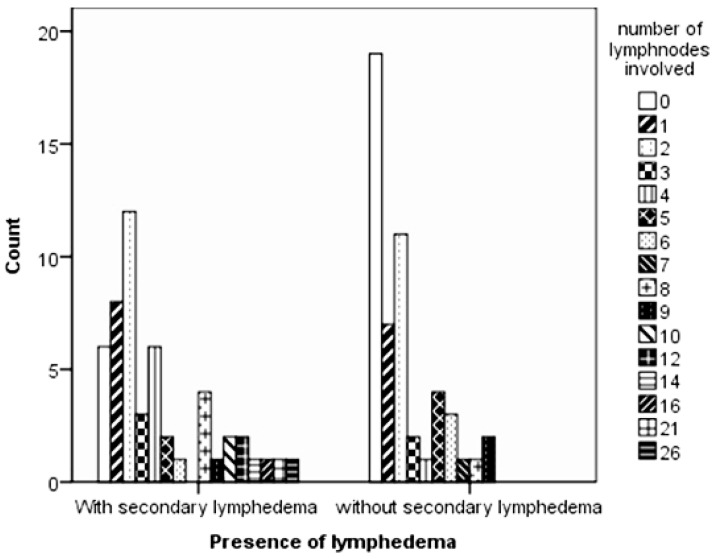
Differences in the number of lymph nodes invaded by tumour cells between the control and case groups.

From the lymph nodes removed, the number of nodes that were invaded by the tumour was assessed (Figure 1); over one-third of the control group had no lymph nodes affected (37.3%), while a similar number in the case group had up to two nodes affected (39.2%). Although the Moses test of Extreme Reaction (nonparametric tests algorithms) revealed no statistically significant difference in the range of the lymph nodes involved (*p* = 0.132), the Mann–Whitney test of distribution indicated a statistically highly significant difference between the control and the case groups (*p* = 0.002).

Binary logistic regression analyses (Table 4) revealed a moderate effects of the tumour parameters on the presence of lymphedema (Cox & Snell R^2^ = 0.231, Nagelkerke R^2^ = 0.309); the overall effect was statistically significant (*p* = 0.001). The multivariate model correctly predicted 72.1% (31 out of 45) of those with secondary lymphedema and 70.6% (36 out of 51) of those without secondary lymphedema; the overall accuracy was 71.3%.

### 3.3. Connexin 37 Genotypes

#### 3.3.1. Melting Profiles

**Figure 2:**
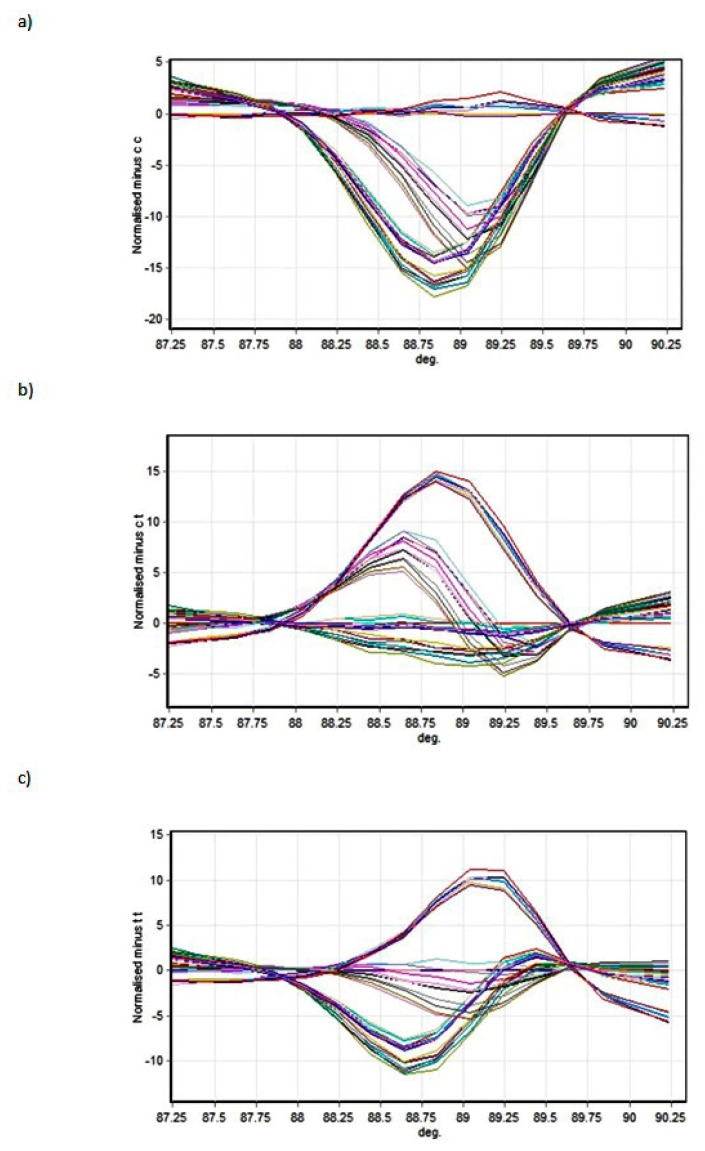
High-Resolution Melting (HRM) detection of rs3543 genotype: (**a**) genotype CC, (**b**) genotype CT, and (**c**) genotype TT. Curve colors represent different samples.

**Figure 3:**
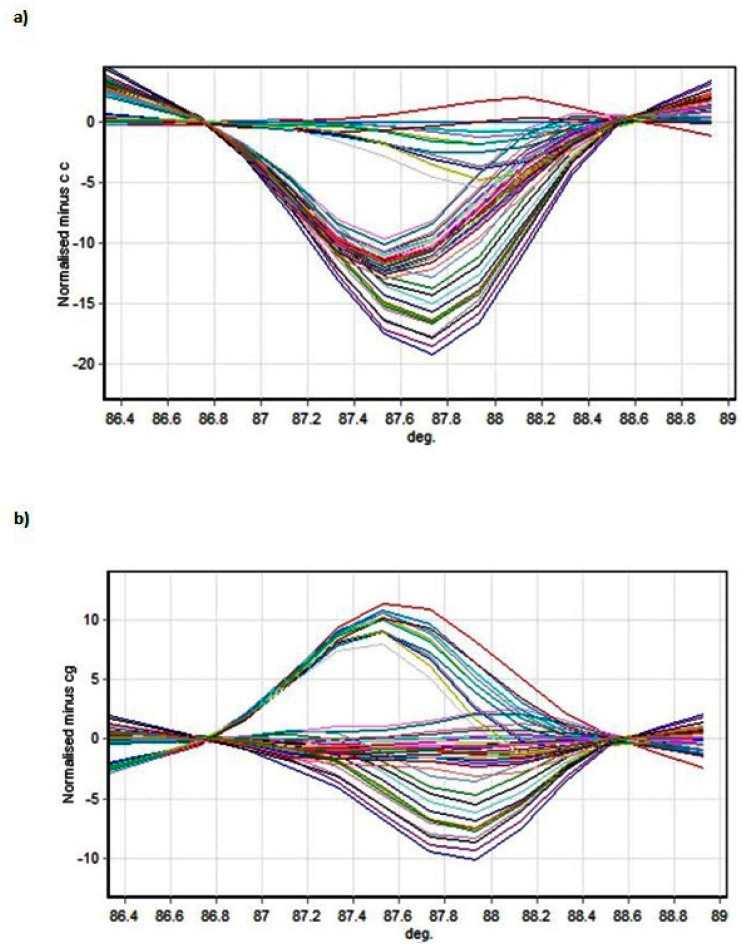
HRM detection of rs705193 genotypes: (**a**) genotype CC and (**b**) genotype CG. Curve colors represent different samples.

Figure 2 and Figure 3 present the melting profiles of rs3543 and rs705193 genotypes, respectively. Homozygous wild-type, mutant, and heterozygote samples are shown on a standard normalized melt curve in Figure 2 and Figure 3. The results for rs3543 show three different melting profiles of analysed amplicons and two melting profiles for rs705193.

#### 3.3.2. Genotypes and Allele Frequencies

Categorical cross-tabulation analyses identified significant associations of allele type T (C→T mutation) with the presence of secondary lymphedema in rs3543 (Table 5). The CC genotype was more abundant in the control group (without lymphedema), while the genotypes CT and TT showed moderate and significant associations with the presence of secondary lymphedema in the case group, respectively. Cramer’s V revealed a medium association which was statistically highly significant (Cramer’s V = 0.385, *p* = 0.001).

Similarly, in rs705193, the C to G mutation contributed significantly to the increased risk of secondary lymphedema (Table 6), while the genotype CG showed significant influence. Cramer’s V indicated a medium association which was highly significant (Cramer’s V = 0.356, *p* = 0.001). 

Interestingly, rs3543 and rs705193 were strongly associated with each other in both the case and the control groups (Cramer’s V 0.803 and 0.819 respectively, *p* = 0.003). The association was not influenced by the allele type TT in rs3543 (−1.96 < z < 1.96, Table 7).

It was evident that for rs3543, the allele type TT had a similar distribution in both the case and the control groups; CT had a small difference between the two groups, and CC had a significant difference between the groups. The absence of the rs3543’s CT and rs705193’s CC combination, together with the lack of the CC and CC combination, contributed to the secondary lymphedema.

## 4. Discussion

The results presented in this paper indicate that two SNPs in the 3’ UTR of the *GJA4* gene are associated with an increased risk of secondary lymphedema in patients being treated for breast cancer. *GJA4* was chosen because it encodes Cx37; other studies have already described that two genes (GJC2 encoding connexin 47 and MET gene) also involved in junction formation have mutations associated with the predisposition to secondary lymphedema [20,21]. The results thus provide strong support for the hypothesis that secondary lymphedema is caused at least partly by genetic factors that presumably lead to inappropriate formation of cellular junctions and, consequently, blockage of the lymphatic system. This has important implications for the diagnosis and treatment of lymphedema.

In comparison to the BCS procedure, although statistically not significant, MRM surgical procedure seemed to increase the odds of secondary lymphedema (odds ratio = 2.766, *p* = 0.075, Table 4). Tumour size, number of lymph nodes removed during the surgery, and number of lymph nodes being invaded by the tumour had little impact on the presence of lymphedema (odds ratio close to 1 and *p* > 0.05). 

The Wald statistic did not indicate that the β coefficients for the genotypes were statistically significantly different from 0 (*p* > 0.05), however the odds ratios for rs3543 (CC) and in particular for rs705193 (CC) showed their odds in favour of without lymphedema (internal value without lymphedema 0, with lymphedema 1).

It is important to note that the SNPs detected are in a region annotated as a 3’UTR, meaning that a direct effect on the protein sequence is unlikely (albeit we have not shown directly that the protein sequence is actually unaffected by the variation, and there remains a possibility that the annotation of this region may be erroneous). Likely, therefore, the mutation associated with secondary lymphedema affects the post-transcriptional fate of the mRNA through effects on stability, as several microRNAs have already been shown to target other connexin family members [22].

Alternatively, there might be effects on transcription through long-range interactions. Finally, it is possible that the variation is functionally insignificant and rather an artefact of linkage or some other confounding variable. Though possible, we consider this latter unlikely in view of the fact that other secondary lymphedema-associated mutations also affect junction-forming proteins [23].

From the molecular pathological point of view, the results presented here suggest that a fruitful approach to secondary lymphedema may be to characterise the cell–cell junctions in healthy and pathological tissues, with the aim of determining, for example, whether the problem is fundamentally linked to junctions that are too tight or too loose [15,16]. Given that the 3’UTR of genes is often involved in RNA stability, we may speculate that the mutations result in loss of function, i.e., less RNA and therefore less protein, which would probably manifest as “too loose” junctions. Alternatively, if the mutations remove a microRNA target, the effect would be increased translation, possibly manifesting as “too tight” junctions. This fundamental and essential work is however beyond the scope of the present study.

The lymphatic drainage pathways of the breast (axillary, internal mammary, and supraclavicular nodal groups) are the regional areas most likely to be involved with metastatic breast cancer, and it has been shown that patients who undergo more extensive surgery, have many lymph nodes removed, or have radiation therapy to the axilla or groin after surgery are more likely to develop lymphedema [24].

The next step of our research, also to increase the strength of our results and conclusion, will be to increase the sample size and to collect similar samples from different geographical areas and other ethnic groups. It is important to notice the importance of ethnicity on the genetic variations and of the sample size, because too big or too small sample sizes have limitations that can compromise the conclusions drawn from studies.

## 5. Conclusions

The results in this study confirm that the number of lymph nodes being invaded by breast tumours had a statistically significant impact on the presence of lymphedema and that increased lymph node invasion correlated with an increased probability of secondary lymphedema.

Significantly, we have discovered a novel predictive biomarker for the predisposition to secondary lymphedema in breast cancer patients, following surgical intervention. Testing for the condition-associated allele should help inform the treatment and post-operative care of patients, with desirable outcomes for the management of breast cancer. Further study of genes involved in junction formation may reveal additional secondary lymphedema-associated polymorphisms, and hence extra biomarkers, offering an exciting new area of breast cancer research.

## Figures and Tables

**Table 1:** Primer sequences and amplicon sizes for rs3543 and rs705193 used in High-Resolution Melting (HRM) analysis.

SNP	Allele	Amplicon Size	Primer
rs3543	C→T	182 bp	Forward: 5′ CTGGAGAGGAAGCCGTAGTG 3′
			Reverse: 5′ CAACAGAGGGGTCCTGAGAA 3′
rs705193	C→G	194 bp	Forward: 5′ CTGATCCAGAGGAACCCAGA 3′
			Reverse: 5′ TGATGAAAACAAGGCACCAG 3′

**Table 2:** Comparisons of physiological measurements and tumour measurements between the control and case groups.

Measurements	Statistics	Significance
Age (year)	*t* = 0.998	*p* = 0.321, *p* > 0.05
Height (cm)	U = 1342.5	*p* = 0.775, *p* > 0.05
Weight (kg)	U = 1150.5	*p* = 0.314, *p* > 0.05
Body surface area (m^2^)	U = 1249.0	*p* = 0.720, *p* > 0.05
Tumour size (cm)		
Control	Median: 5; Lower: 4; Upper 5 *	*p* = 0.277
Case	Median: 5; Lower: 5; Upper 5 **	*p* = 0.618
No. of lymph nodes removed		
Control	Median: 10; Lower: 8; Upper 12 *	*p* = 0.132
Case	Median: 10; Lower: 9; Upper 10 **	*p* = 0.997
No. of lymph nodes involved		
Control	Median: 1; Lower: 0; Upper 2 *	*p* = 0.132
Case	Median: 2; Lower: 2; Upper 4 **	*p* = 0.002

* The range is the same; ** The distribution is the same.

**Table 3:** Effects of age, height, weight, and body surface area on the presence of secondary lymphedema.

Physiological Parameters	Age (Year)	Height (cm)	Weight (kg)	Body Surface Area	Constant
β	−0.027	0.028	−0.030	−0.618	−0.247
S.E. *	0.026	0.039	0.023	10.118	50.856
Wald **	10.034	0.533	10.593	0.306	0.002
Sig.	0.309	0.465	0.207	0.580	0.966
Odds ratio Exp (β)	0.974	10.029	0.971	0.539	0.781
95% CI *** for odds ratio					
Lower	0.924	0.953	0.927	0.060	
Upper	1.025	1.110	1.017	4.820	

* S.E., the standard error around the coefficient for the constant; ** Wald, the Wald chi-square test; *** CI: Confidence Interval.

**Table 4:** Effects of clinical parameters and genotypes assessed on the secondary lymphedema.

Clinical Parameters and Genotypes	*b*	S.E.	Wald	*p*	Odds Ratio	95% CI for Odds Ratio	
						Lower	Upper
Surgery Method (MRM)	1.017	0.572	3.162	0.075	2.766	0.901	8.488
Tumour size (cm)	0.050	0.130	0.149	0.699	1.052	0.815	1.357
No. of lymph nodes removed	−0.089	0.076	1.385	0.239	0.914	0.788	1.061
No. of lymph nodes involved	0.150	0.099	2.277	0.131	1.162	0.956	1.411
rs3543 (CC)	−1.570	1.037	2.296	0.130	0.208	0.027	1.586
rs3543 (CT)	−0.351	0.637	0.304	0.582	0.704	0.202	2.454
rs705193 (CC)	−1.025	0.931	1.212	0.271	0.359	0.058	2.226
Constant	0.275	1.316	0.044	0.835	1.316		

**Table 5:** Cross-tabulation analysis of rs3543 allele frequencies in the case and the control groups.

Presence of Secondary Lymphedema		rs3543			Total
		CC	CT	TT	
**With secondary lymphedema (Case group)**	Count	5	31	15	51
	% within Presence of lymphedema	9.8%	60.8%	29.4%	100.0%
	% within rs3543	18.5%	58.5%	68.2%	50.0%
	Std. Residual (z)	−2.3	0.9	1.2	
**Without secondary lymphedema (Control group)**	Count	22	22	7	51
	% within Presence of lymphedema	43.1%	43.1%	13.7%	100.0%
	% within rs3543	81.5%	41.5%	31.8%	50.0%
	Std. Residual (z)	2.3	−0.9	−1.2	
**Total**	Count	27	53	22	102
	% within Presence of lymphedema	26.5%	52.0%	21.6%	100.0%
	% within rs3543	100.0%	100.0%	100.0%	100.0%

**Table 6:** Cross-tabulation analysis of rs705193 allele frequencies in the case and the control groups.

Presence of Lymphedema		rs705193		Total
		CC	CG	
**With secondary lymphedema (Case group)**	Count	8	43	51
	% within Presence of lymphedema	15.7%	84.3%	100.0%
	% within rs705193	24.2%	62.3%	50.0%
	Std. Residual (z)	−2.1	1.4	
**Without secondary lymphedema (Control group)**	Count	25	26	51
	% within Presence of lymphedema	49.0%	51.0%	100.0%
	% within rs705193	75.8%	37.7%	50.0%
	Std. Residual (z)	2.1	−1.4	
**Total**	Count	33	69	102
	% within Presence of lymphedema	32.4%	67.6%	100.0%
	% within rs705193	100.0%	100.0%	100.0%

**Table 7:** Association of rs3543 and rs705193 in the case and the control groups.

Presence of Lymphedema		rs3543			Total
		CC	CT	TT	
With secondary lymphedema (Case group)	rs705193 CC Count	5	0	3	8
	Std. Residual (z)	4.8	−2.2	0.4	
	CG Count	0	31	12	43
	Std. Residual (z)	−2.1	1.0	−0.2	
	Total Count	5	31	15	51
Without secondary lymphedema (Control group)	rs705193 CC Count	21	2	2	25
	Std. Residual (z)	3.1	−2.7	−0.8	
	CG Count	1	20	5	26
	Std. Residual (z)	−3.1	2.6	0.8	
	Total Count	22	22	7	51
Total	rs705193 CC Count	26	2	5	33
	Std. Residual (z)	5.8	−3.7	−0.8	
	CG Count	1	51	17	69
	Std. Residual (z)	−4.0	2.5	0.5	
	Total Count	27	53	22	102

## Supplementary Materials

The Physiological and tumour parameters (.xlsx) are available online at http://www.mdpi.com/2227-9059/6/1/23/s1.
